# Twenty years (2000–2020) of butterfly monitoring data across the contiguous United States

**DOI:** 10.1038/s41597-025-05513-8

**Published:** 2025-11-22

**Authors:** Erica H. Henry, Collin B. Edwards, Vaughn Shirey, Jeffrey S. Pippen, Dave Waetjen, Matthew L. Forister, Elise A. Larsen, Cheryl B. Schultz, James Michielini, Nathan Brockman, Kevin J. Burls, Ryan G. Drum, Martha Gatch, Jeffrey Glassberg, Nancy V. Hamlett, Shiran Hershcovich, Catherine Le, Steve McGaffin, Jen Meilinger, Lisa Richter, Regina Rochefort, Charles Schelz, Arthur M. Shapiro, Kathryn Sullivan, Doug J. Taron, Wayne E. Thogmartin, Anna Walker, Anita Westphal, Jerome Wiedmann, Irmgard U. Wilcockson, Jennifer Zaspel, Leslie Ries

**Affiliations:** 1 School of Biological Sciences, Washington State University, Vancouver, WA USA; 2 Washington Department of Fish and Wildlife, Washington, USA; 3 Biology Department, Georgetown University, Washington, DC USA; 4 Road Ecology Center, University of California, Davis, CA USA; 5 Department of Biology, University of Nevada, Reno, NV USA; 6 Department of Evolution and Ecology, University of California, Davis, CA USA; 7 Reiman Gardens, Iowa State University, Iowa, USA; 8 Nevada Bugs and Butterflies, Nevada, USA; 9 Center for Pollinator Conservation, United States Fish and Wildlife Service, Washington, DC, USA; 10 Massachusetts Butterfly Club, Massachusetts, USA; 11 North American Butterfly Association, New Jersey, USA; 12 Partnership for Regional Institutions for Sage Scrub Monitoring, California, USA; 13 Butterfly Pavilion, Colorado, USA; 14 Irvine Ranch Conservancy, California, USA; 15 Zoo Knoxville, Tennessee, USA; 16 Kalamazoo Nature Center, Michigan, USA; 17 Missouri Department of Conservation, Missouri, USA; 18 North Cascades National Park, Washington, USA; 19 Cascade-Siskiyou National Monument, Oregon, USA; 20 Center for Population Biology, University of California, Davis, California USA; 21 Milwaukee Public Museum, Wisconsin, USA; 22 Chicago Academy of Sciences, Illinois, USA; 23 U.S. Geological Survey, Upper Midwest Environmental Sciences Center, Wisconsin, USA; 24 New Mexico BioPark Society, New Mexico, USA; 25 Ohio Lepidopterists Society, Ohio, USA; 26 Texas Butterfly Monitoring Network, Texas, USA

**Keywords:** Biodiversity, Population dynamics

## Abstract

We present the most comprehensive, integrated, butterfly monitoring dataset ever assembled for the United States. It contains over 1.2 million count records, from 65,000 surveys, representing over 12.6 million individual butterflies. To compile this dataset, we integrated data and harmonized taxonomy across 19 butterfly monitoring programs in the United States – one national, 13 statewide, and 5 local (e.g. individual county or National Park) in scale. In addition to the data, we also provide the taxonomic dictionary used to crosswalk butterfly taxonomy across programs, and the code used to assemble the integrated dataset. The publication of this dataset will inspire new analyses of butterfly population trends and drivers that help to identify solutions to the biodiversity crisis.

## Background

Tracking trends in biodiversity and species abundance at continental scales requires long-term monitoring across time and space. Ideally, monitoring involves spatiotemporally replicated structured surveys with similar protocols to support granular time series analyses^1^. Data from these types of surveys have the power to detect trends and explore putative drivers of trends. This kind of monitoring is especially difficult for insects (and other invertebrates) because (1) they are incredibly diverse, requiring substantial field training, and (2) have short generation times and high annual reproductive output creating highly variable population dynamics. For insects, ideal monitoring includes multiple surveys per-year to capture annual phenology of entire (or multiple) generations which requires high amounts of effort^2^. Compared to all other insect taxa (not including individual pest species), butterflies are the most extensively studied and monitored making them the best candidate for identifying trends in abundance over time.

The United States is home to some of the largest and longest-term butterfly monitoring programs in the world. Indeed, the longest-term monitoring program in the United States (and one of the longest-running in the world) is Dr. Arthur Shapiro’s^3^ (https://butterfly.ucdavis.edu/). Starting in 1972, Dr. Shapiro began establishing a set of transects across Northern California that have been monitored every other week during the butterfly flight season for between 37 and 53 years, depending on the site; most data were collected by Shapiro, with more recent years involving collaboration with Forister. In 1975, the Xerces Society for Invertebrate Conservation started the first continental-scale butterfly monitoring program. That program, now led by the North American Butterfly Association (NABA)^4^, is the largest, longest-running volunteer butterfly monitoring program in the world^5^. In the decades that followed, local-to-regional programs were established to monitor butterfly population dynamics more closely, and those programs, many state-wide, have been steadily growing in the US. The first of these regional monitoring programs began in Illinois in 1987, and now there are at least 25 local-to-regional programs (cataloged at https://thebutterflynetwork.org/) with new ones being established (e.g., Carolinas Butterfly Monitoring Program in 2023 and the Blue Ridge Butterfly Monitoring Program in 2024) and older ones being discovered regularly^5^.

Despite this wealth of butterfly data in the US, true integrated analyses of species’ national trends have been rare. Single-species butterfly trends have only been estimated within a single state with data from a single program^6–10^, or at larger (regional, national) spatial scale with data from a single monitoring program^11,12^, or at large spatial scales but for community-only metrics, specifically overall biomass or species richness^12,13^. More comprehensive analyses have been stymied by the difficulty of integrating data from different programs with variable protocols and differing taxonomic standards, but recent advancements in statistical modeling and improvements in interoperability^14,15^ have lowered those barriers. Recent examples of data integration across butterfly programs, where there is a single analysis with all data sources included, are rare for the US and Europe and, to our knowledge, include only Zylstra *et al*.^16^, Farr *et al*.^17^, and Edwards *et al*.^18^. The strength of an integrated analysis is that results are less subject to potential regional or programmatic biases than when data sources are analyzed individually^19^. These large-scale trend analyses are vital if we are to pinpoint potential causes and solutions to the biodiversity crisis^20^.

A key limitation to an integrated analysis of all butterfly data in the US is that each data source has unique qualities even if they follow the same protocols. This arrangement means that, despite similarities, data must be compiled and formatted into standardized data structures in such a way that allows for integrated analyses (see *Data Harmonization* below). Most of the monitoring data in the US are collected by dedicated volunteers with minimal funding and capacity for ensuring data are compatible across programs. Instead, data compilation can fall to individual researchers who may not be familiar with the nuances of each program’s dataset and who must independently align disparate taxonomic and data collection frameworks, risking the loss or misinterpretation of the original meaning of the data.

The dataset shared with this publication is the most comprehensive butterfly monitoring dataset assembled to date. Starting in 2022, with support from the USGS Powell Center for Analysis and Synthesis and the United States Fish and Wildlife Service, a multidisciplinary group of scientists (Status of Butterflies of the United States Working Group, hereafter SBUS) convened with the goal of assembling a nation-wide butterfly monitoring dataset with which to evaluate status and trends of US butterflies. The group’s effort resulted in an integrated dataset, compiled from 35 monitoring programs, and trend estimates for 342 butterfly species^18^. The full Edwards *et al*. integrated dataset included programs that target endangered species monitoring and programs that monitor the butterfly community. Here, we present the integrated SBUS dataset, excluding endangered species monitoring datasets due to data sharing restrictions^21^, and the steps taken to integrate disparate monitoring programs while honoring individual program data sharing policies. This dataset integrates data from 19 butterfly community monitoring programs, representing 64,984 surveys and 12.6 million individual butterfly observations.

## Methods

### Data types

We included butterfly monitoring data from three types of butterfly surveys detailed below: circle counts, field trips, and repeated transects.

#### “Circle” counts (*NABA Seasonal Counts*)

Teams of observers conduct a one-calendar-day complete count of all adult butterflies at various sites within a pre-established and non-varying 24-km diameter “Count Circle”. Because most counts are near mid-summer, these have previously been known as the “Fourth of July” butterfly counts. Effort is recorded as “party hours”, the sum of the number of hours each group of participants was in the field surveying. Total number of participants in each party and total miles walked by each party while surveying are also recorded. Data collected in these surveys are almost entirely collected by volunteer public scientists. Counts are conducted across the country.

#### Field trip (*Massachusetts Butterfly Club - a NABA chapter*)

Participants on organized field trips identify and record butterflies seen with some individuals reporting opportunistic sightings. Sometimes species are counted and tallied, but that is not always the case. No measure of effort is systematically recorded across all field trips. Data are almost all collected by volunteer public scientists. Counts are restricted to Massachusetts.

#### Structured “transect” surveys (*PollardBase programs, Shapiro data*)

Observers conduct repeat (usually weekly or biweekly) visits to transects throughout the warm-weather flight season. Most of these use “Pollard Walk” methods in which observers walk transects and count and identify all butterflies encountered within a 5-meter forward radius of the observer (180 degrees). Most programs report time spent by observers on each survey, as well as transect lengths as a measure of effort. Data in PollardBase programs are largely collected by volunteer public scientists, while the Shapiro dataset is collected by researchers. Counts in these surveys depend on the focal extent of each program (see Supplementary Table 1).

### Individual data sets

**Fig. 1:**
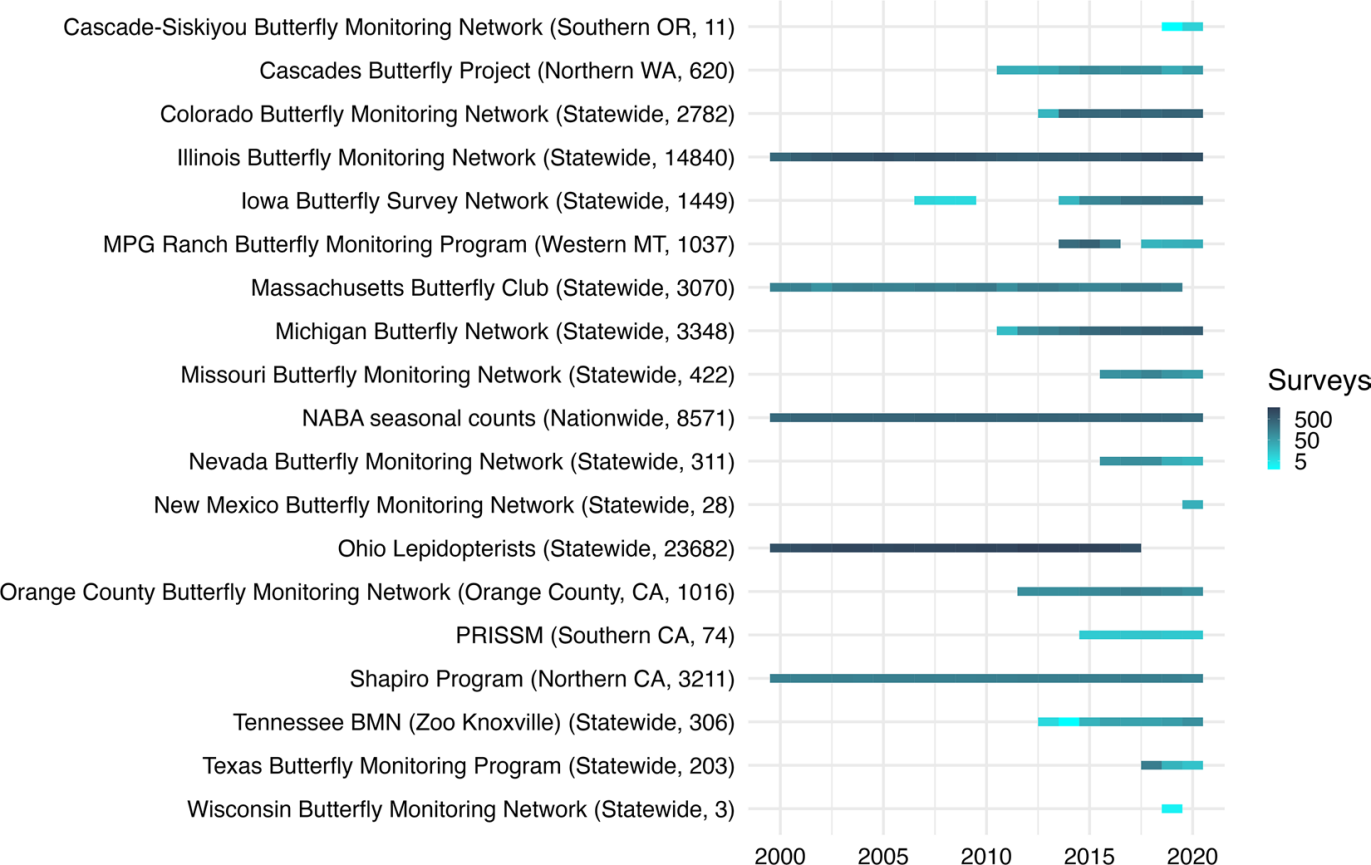
Data metrics for 19 programs integrated in the SBUS dataset. Program extent and number of surveys are in parentheses; the number of surveys in each year is indicated by color.

The final integrated dataset includes data from 19 butterfly monitoring programs – one national, 13 statewide, and 5 local (e.g. individual county or National Park) in scale (Fig. 1). Data are collected by volunteer observers for all programs except the Shapiro program. The Shapiro data are collected primarily by Dr. Shapiro as well as a few other professional scientists. Details of each program’s data, spatial and temporal resolution, and data sharing requirements are given in Supplementary Table 1. The number of years and number of surveys varies across the programs. Five programs began collecting data prior to 2000 (the earliest year included in the dataset), the rest of the programs began collecting data across the last two decades, the most recent of which began in 2020 (Fig. 1). The total number of surveys contributed by each program ranges from 3-23,000 (Fig. 1). For three programs, Ohio, Massachusetts, and Wisconsin, data up through 2020 had yet to be entered and uploaded at the time of data integration and analysis; these programs continue to operate.

### Harmonize taxonomy

Not all data providers use the same taxonomic standard (Supplementary Table 1). Most programs use The NABA Checklist of English Names of North American Butterflies^22^ or A Catalogue of Butterflies of the United States and Canada by Jonathan Pelham^23^, also known as “The Pelham Catalogue”, while a few programs base their species list on the Scientific Names List For Butterfly Species of North America, North of Mexico^24^. One of the main differences between the three butterfly taxonomies is that NABA generally does not recognize subspecies while the other two do. In addition, NABA is more reluctant to split one species into several (usually through elevating sub-species to species status). In essence, NABA are “lumpers” and Opler-Warren and Pelham are “splitters” who are quicker and more likely to adopt results from recent scientific publications affecting taxonomy. To complicate things further, some programs initially downloaded state lists from Butterflies and Moths of North America (BAMONA; butterfliesandmoths.org), and have tailored these lists over the years. BAMONA tries to represent the most widely accepted taxonomy, using both Opler-Warren and Pelham lists as sources. They acknowledge “in this ever-changing realm of taxonomy, we are not always up to date, and some names are always in dispute.” Each program also has individual nuances in terms of which butterflies they allow observers to differentiate. For example, some programs do not allow observers to differentiate Orange Sulphur (*Colias eurytheme*) from Clouded Sulphur (*Colias philodice*) due to the quality of views and level of experience needed to make the identification in the field.

The taxonomic variation across programs makes harmonizing taxonomy the necessary first step to integrating datasets. Because the NABA checklist is the coarsest of the taxonomic standards used (ie. few subspecies are included), we standardized taxonomy across all programs to the NABA list, based on a framework developed for the North American Butterfly Monitoring Network (NABMN)^25^. Essentially, we gave each NABA species a six-letter code and created a dictionary to crosswalk species names from all programs to their corresponding six letter code. For example, if an observer detected a Fender’s blue butterfly, a threatened subspecies in Oregon, a program with a NABA taxonomic standard would record that butterfly at the species level, *Plebejus icarioides*, but a program using the Pelham taxonomy, would recognize the subspecies and record that butterfly as *Plebejus icarioides fenderi*. For the purposes of harmonizing the taxonomy across programs, in our six-letter code system both of these records would feed into the code PLEICA. The codes and the species associated with them are updated as necessary to accommodate changes to the various taxonomic standards. In this data release, we preserved many species names as reported so that higher taxonomic resolution is preserved, but data integration is still possible. In this process we also resolved many spelling errors encountered in the raw data. To allow future efforts to integrate datasets, we include our taxonomy dictionary in this data release.

### Anonymize data

As part of developing data sharing agreements with monitoring program directors, we established the spatial and taxonomic resolution at which they were willing to share records in a data release and whether to share actual site names or have them anonymized. To streamline anonymization and data re-integration, we provided program directors the option of sharing location data as the raw lat/long, lat/long rounded to the nearest 0.1 degree (approximately + /−5–10 km), or rounded to the nearest full degree (approximately + /− 100 km). For taxonomic resolution, all programs agreed to share species names as reported. This decision means that if programs report species as a trinomial but taxonomic harmonization is binomial, the original species name is preserved in the data set as it was reported. No program provided observer names, so individual observers are not identified in this dataset.

## Data Records

**Fig. 2:**
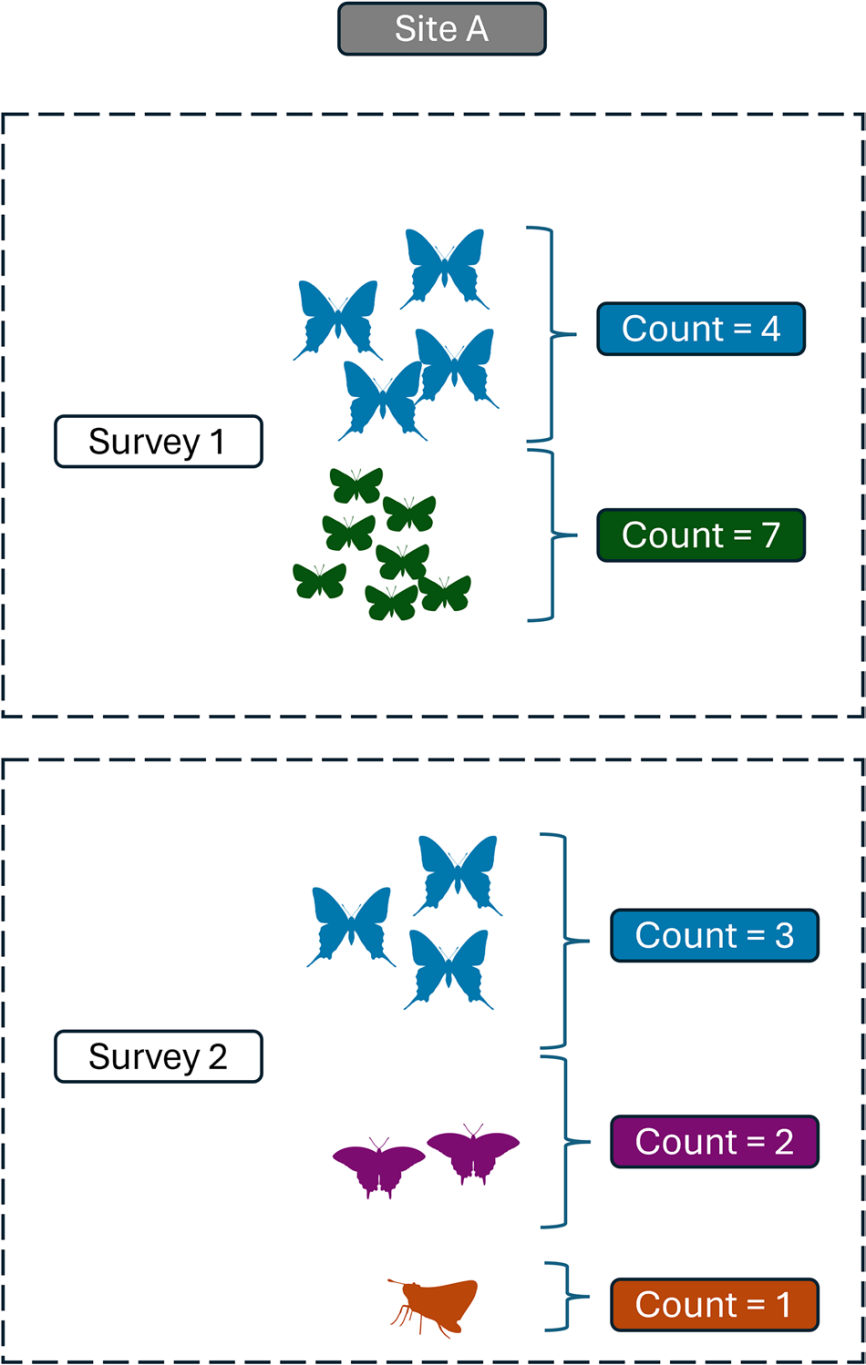
An example site that is surveyed two times. Each survey is given a unique event.id. Survey 1 would be represented in the dataset as two records, one for each species detected in that survey with its respective count, survey 2 would be represented by three records. In this case, five records represent a total of 17 butterflies observed.

The final SBUS dataset includes 1.4 million data records. Each record consists of a butterfly species count (can be >1) from an individual survey (Fig. 2) along with information about the site identity (including latitude and longitude, anonymized as requested, Table S1), survey date, and, when available, information about survey effort. Sites generally represent unique locations that were visited for multiple survey events, each survey event is given a unique event ID. The final dataset spans 2000–2020, the period over which Edwards *et al*.^18^ estimated abundance trends.

We present the dataset and the code used to generate it in FigShare: 10.6084/m9.figshare.27934602. This directory is an R project, and details of the directory structure and files are available in “Code map.txt”. We describe the directory structure and key files in order below; details on the primary data files are found in the description of “4_res/”. The complete version of this directory contained survey data with non-anonymized details; we have removed those files, but retained the file structure so that if someone obtained permission to use the original data files, they could place those files in “1_raw_data” and then recapitulate our data cleaning pipeline by running the file “3_scripts/clean_run.R”.

The folder “1_raw_data” contains the table of program permissions; in the complete version of this directory, this folder also contains the original survey data. However, that survey data included non-anonymized information, so those files were removed before uploading to Figshare.

The folder “2_data_wrangling/” contains the “dictionaries/” folder, which contains files to harmonize taxonomy errors by mapping reported scientific names to the NABMN naming convention. “2_data_wrangling/dictionaries/LeslieSpeciesCodes-reconciled.csv” contains the most up to date mapping between scientific names listed in monitoring data (column ‘ScientificNameasReported‘) and NABMN code (column ‘UMD-CODE‘). “2_data_wrangling/dictionaries/LeslieSpeciesCodes.Oct.2022.csv” contains additional taxonomic details for each reported scientific name, including ‘Family‘, ‘Subfamily, ‘Genus‘, ‘Species‘, ‘Subspecies‘, and ‘Common Name‘ for each butterfly. The ‘Order’ column is a unique identifier for each reported scientific name. Column ‘TaxLevel‘ identifies what taxonomic level the butterfly is identified at, and ‘IncludeSubspecies‘ identifies the four subspecies which are handled at the subspecies level in the NABMN codes: *Boloria improba acrocnema, Limenitis arthemis arthemis, Limenitis arthemis astyanax*, and *Lycaeides melissa samuelis*. The folder “2_data_wrangling/” also contains “FWS-regions-by-state.csv” which identifies the Fish and Wildlife geographic region (‘region‘) associated with each state in the contiguous united states plus the District of Columbia (‘state‘). The complete version of this folder contained processed data in ‘cleaned-data‘ and ‘data-deduplication‘, but those files were removed to protect the anonymity of data.

The folder “3_scripts/” contains the scripts used to process the original data. When the (removed) non-anonymized data is present, script “clean-run.R” can be run to completely rerun the entire process from cleaning the original data to producing the anonymized data files and the figures used in this publication. However, only the script for data generation will work without access to the non-anonymized data; we have added conditional statements of ‘if(FALSE)‘ around the sections that depend on removed data. The scripts in subfolder “data-cleaning-scripts/” process the original data survey data files, remove duplicates, and produce a single non-anonymized csv file; the function “program-processing-and-anonymization.R” then appropriately anonymizes each program’s data and saves an overall file and program-specific files into the folder “4_res/” (details below). The script “data-figures-eh.R” generates the figure used in this publication and saves it to “5_figs/” (details below). “funs.R” contains R functions used in data processing.

The folder “4_res/” contains the anonymized data files. “complete-share.csv” contains all survey data, anonymized appropriately. Each row represents observations of one or more individuals of a single species in one survey. The columns are as follows:

- ‘program‘: butterfly monitoring program
- ‘region‘: One of seven Fish and Wildlife regions (“Pacific” has been relabeled “Pacific Northwest” for clarity).
- ‘state‘: U.S. state (or District of Columbia)
- ‘year‘: year of observation
- ‘date‘: date of observation
- ‘doy‘: day of year as a numeric
- ‘site‘: site identity, with site name anonymized if requested by program.
- ‘event.id‘: survey identifier. All rows sharing an event.id came from the same survey. Original event.id values often contained site names within them; event.ids were anonymized when programs requested site be anonymized.
- ‘Reported species name‘: species name as provided in the survey
- ‘GU Code‘: NABMIN code used to harmonize taxonomy.
- ‘count‘: number of butterflies seen
- ‘lat-lon-precision‘: the level of anonymization that was applied to latitude and longitude, based on program request
- ‘lat‘: latitude, anonymized to the specifications of ‘lat-lon-precision‘
- ‘lon‘: longitude, anonymized to the specifications of ‘lat-lon-precision‘
- ‘duration‘: Minutes spent surveying (for programs other than Naba Circle Counts)
- ‘party.size‘: number of people in the survey group (Naba Circle Counts only)
- ‘party.minutes‘: person-minutes spent by survey group (Naba Circle Counts only)

In addition to the flat file with all data, subfolder “individual share/” contains the same information in a separate excel file for each program. Within those files, sheet “Data” has the same structure as described for “complete share.csv” above, and the other two sheets contain summary information on the number of sites, survey, species, and individual butterflies seen overall (“Basic data info”) or by year (“Yearly data info”). In the complete folder, subfolder “individual actual/” contained non-anonymized data in a file for each monitoring program.

The folder “5_figs/” is the location for figures. Running “scripts/data-figures-eh.R” creates the figure in this publication and saves it here.

## Technical Validation

### For each individual program

The harmonized data presented in this paper comes from several independent butterfly monitoring programs and each program has its own process for technical validation of their data. Of the programs included in this data paper, validation is aligned with the data management system for each program. The 19 programs included in this data are managed under four different systems:

- The **Shapiro program** is unique in that all the data are part of an academic project and collected by the same person (Art Shapiro, except for high altitude sites starting in year 2018). The small group of people associated with data collection and data management (personnel associated with either Art Shapiro or Matt Forister’s labs) mean that data collection protocols and quality assurances are maintained at a higher level of rigor than is possible for other programs. Specifics of data collection and management have been described elsewhere^3^. In short, field observations are recorded into a formal field notebook, which are all archived. Data are then entered into a database by lab personnel and queried for unusual records. Any flagged records are double checked and, if necessary, a query is sent to Shapiro to confirm the record. Finally, data are then quality checked by lab personnel against the original forms and this checked database is sent back to Shapiro or Forister for a final quality assurance check.
- The **Ohio Lepidopterist program** is managed by Jerry Wiedmann and, until 2020 (when closed because of Covid), the Cleveland Museum. After 2020, all data management is done by Wiedmann. Data forms are filled out by individual volunteers and then sent for data entry. Data are entered into an Access database and records are quality checked for entry and unusual records by Wiedmann.
- The **NABA Seasonal Count program** is managed by the North American Butterfly Association. This nationwide program has two checks on the validity of the data. First, at the end of most counts, parties gather to combine their data and, at that time, any unusual records can be vetted by that count circle’s lead. Data are then entered directly into a data portal where they are then checked by regional coordinators who also vet the data for unusual records or obvious mistakes. Data are then accepted into the main data warehouse that is managed by NABA.
- All other programs included in this data set are managed in an online data platform called **Pollardbase**. Each program has its own “instance” of the system, so all processes and data are completely siloed for each program. Although different programs have slightly different data handling protocols, they all follow the same basic blueprint. Data are collected by individual volunteers and those volunteers enter their own data into the system. When a survey is entered, it triggers a notification to the program director (or regional directors for programs that have those). The directors review each survey, determine if there are any unusual sightings or apparent mistakes, then they contact the person who performed the survey to check the validity or make corrections as appropriate. These directors also assess surveys for any indication that program protocols were not followed. If not, the data are flagged as “non-conforming”, but still are entered into the database. Non-conforming surveys are excluded from any shared datasets unless explicitly requested; no non-conforming surveys are included in the dataset described in this paper.

### For this project’s database

After harmonizing the data, we took several steps to ensure the quality and usability of the data. We started by removing incomplete observations, for example those that did not include latitude, longitude, or the number of butterflies seen. This step included removing records from the Shapiro data that only provided presence-absence data; these records can be obtained from https://butterfly.ucdavis.edu/. In some records, the given US state did not correspond to the reported latitude and longitude. For consistency, we used the reported latitude and longitude to assign each survey to the appropriate state. Some programs create records separately for each section of a survey, meaning that a single survey can have multiple records for a single species. We summed these counts to obtain a single count per species per survey. Finally, we checked for duplicate records in our dataset. In this process we found pairs of surveys with different latitude/longitude values. This generally was explained by different rounding decisions, we kept the record with the more precise location and removed the corresponding duplicate. We also found pairs of surveys with identical contents but different reported survey effort. For these, we averaged the effort of the two records and removed duplicates.

## Usage Notes

Prior to assembling the integrated dataset, we developed individual data sharing agreements with each independent butterfly monitoring program (See Supplemental Information for example agreement template). Our primary goal for these agreements was to ensure that we adhered to data privacy needs of each program. The dataset conforms to spatial resolution and anonymization standards stipulated in each program’s data sharing agreement (Supplemental Table 1).

Although the dataset, as we have shared it, is available to users without additional data sharing agreements, we strongly recommend users of the data contact Program Directors when developing a project with this dataset. Contacting data owners is a best practice when using accessible data for two main reasons: 1) By communicating with data owners, researchers help facilitate ongoing data collection efforts. Program directors organize volunteers, coordinate and facilitate data entry, maintain relationships with local landowners where counts are conducted, and fundraise to support their programs, among many other tasks. Being able to track who is using their data and for what purposes is key to sustaining their efforts in the long term. 2) Datasets are complex and the details matter. From both methodological and biological perspectives, data owners will often have useful information that is not necessarily captured in the metadata attached to this publication. For example, consultation with the data owner might reveal that an apparent decline in the data, for a particular species, is happening in the context of shifting metapopulation dynamics rather than local or regional extirpation; that knowledge would not affect analyses but should affect interpretation. As another possibility, a data owner might know that taxonomic identification within some particular species complex is difficult and could introduce uncertainty to trends over time. It is also the case that indices of effort are best handled in dataset-specific ways, and data owners will have a knowledge of this and can advise; for example, if a measure of effort is better treated as an offset or as a linear or quadratic covariate. Communication with data owners provides continuity on best practices for these and other issues.

A number of programs opted to share data at a coarsened spatial resolution or anonymized site names (Supplemental Table 1). We provided these programs with a raw data file (not anonymized) that is formatted to be easily integrated with this dataset, the taxonomy is harmonized and the columns are standardized. These raw data files are available from individual programs upon request and completion of a data sharing agreement. Additionally, as data are added to these datasets each year, each monitoring program is equipped to provide an updated dataset if/when that request is made. Contact information for each program director is included in Supplemental Table 1.

We encourage users of the data to consider and account for different program data collection methods in their analyses. We have outlined several key nuances here and, more broadly, encourage careful thought and outreach to the individual programs as necessary. Effort metrics must be included in analyses of these datasets, given that it can be highly variable across programs and sites^11,18,26^. For example, this dataset includes some observations in which observer effort is reported but perhaps improbable: less than or equal to zero minutes, or greater than 10 hours continuous observation time, Edwards *et al*. removed these observations for analyses. Similarly, interpreting butterfly counts directly can require care, as some programs report 0 individuals seen for absent species (e.g., Shapiro) while others only report species when present; Edwards *et al*.^18^ imputed the implied zeroes from presence-only programs in order to avoid the clear bias of missing 0 s from some programs. Additionally, the meaning of “site” can vary subtly between programs, and lead to different levels of comparability among programs. Surveys at the same site will be highly comparable for programs that use structured transect surveys as the same route is walked every time, while two surveys in the same NABA circle count may have been several miles apart.

## Supplementary information

Supplementary Table 1

Supplementary Information

## Data Availability

Code used to generate the figure in this manuscript, as well as all code used to assemble the integrated dataset is available in the FigShare^27^ repository.
