# Supplementary material for: Twenty years (2000–2020) of butterfly monitoring data across the contiguous United States
Source: Sci Data. 2025 Nov 22;12:1869. doi: 10.1038/s41597-025-05513-8 (PMC12660994; doi:10.1038/s41597-025-05513-8)
Supplement: Supplementary file 2 — Supplementary Information [file 41597_2025_5513_MOESM2_ESM.docx]

**Memorandum of Understanding**

**Data Sharing Agreement /Memorandum of Understanding for Data Use**

Between (Data Provider): ________________________ Butterfly Monitoring Program

And (Data Requestor): ______________________________________________________

This Memorandum of Understanding (MOU) sets forth the terms and understanding between the above mentioned partners for data sharing and data use.

**Background**

The ______________Butterfly Monitoring Program run by the _______________________ (Organization) uses citizen science to conduct butterfly surveys across its region. Data are collected by volunteers and/or paid researchers and are deposited in the international web-based PollardBase data site (<https://www.pollardbase.org/>).

PollardBase is a data entry and management system for butterfly monitoring programs that follow specific protocols for monitoring butterfly activity along known routes or transects. PollardBase is supported in part by an award from the US National Science Foundation to the University of Maryland. PollardBase is currently supported by Georgetown University.

**Purpose**

This MOU will govern the conditions under which the Data Requestor may use the data gathered by the Butterfly Monitoring Program (Data Provider). If applicable, it is at the discretion of the program director to instruct the administrators at PollardBase to provide data to the Data Requestor as described in this Agreement. The specific request for data is attached to this agreement (Appendix A).

**Provisions**

- Data are generally provided at the level of _______________. If a different (lower/higher) degree of data granularity is requested, please provide details. The Requestor does not assert any intellectual property rights for the data that are made available by the Program.
- Any requests for an update of data require a new MOU.
- The Data Provider makes reasonable efforts to ensure that the data they provide are accurate and up to date.
- Responsibility regarding the restriction of access to sensitive data (such as sites harboring Endangered Species) resides with the Data Provider. Specifically, we do not provide the following:
  - Access to [list of sensitive species], unless specified in this MOU
  - Access to [any particular site], unless specified in this MOU
- The Data Provider is not liable or responsible, nor are its employees, contractors, or volunteers, for the subsequent use and interpretation of the data by the Requestor.
- The Requestor will provide a statement of acknowledgment in any peer-reviewed publications acknowledging the Program providing the data [and funding source if applicable] and PollardBase [including reference to NSF funding ID: NSF-1738243]. You may also request that volunteers who collect data are acknowledged as a group.
  - Authorship for Program participant should be considered if… []
- The Data Requester will maintain confidentiality of the data provided and will not further share the data outside of the current project covered by this Agreement.
- The Data Requester will notify the Data Provider within 30 days of publishing scientific or technical papers or other documents (for example, report to conservation organization or government agency) using data derived from the Data Provider and will provide a copy of the paper or report.

**Financial Arrangements**

This agreement does not require any exchange of funds. If a situation arises whereby funds exchange is required, a separate document will be prepared to specify the terms.

**Duration**

This MOU is [____] and may be modified by mutual consent of authorized officials from the parties mentioned at the beginning of this document (Data Providers and Data Requestors). This MOU shall become effective upon signature by the authorized officials from both parties and will remain in effect until modified or terminated by any one of the partners by mutual consent.

**Contact Information**

Requestor Name/Project:

Requestor Representative:

Position:

Address:

Telephone:

Fax:

E-mail:

Requestor Signature: _________________________________ Date: ___________________

Provider Name/Project:

Provider Representative:

Position:

Address:

Telephone:

Fax:

E-mail:

Provider Signature: _________________________________ Date: ___________________
